# Evaluation of involved proteins in colon adenocarcinoma: an interactome analysis 

**Published:** 2017

**Authors:** Reza Valizadeh, Ayad Bahadorimonfared, Mostafa Rezaei-Tavirani, Mohsen Norouzinia, Mohammad Iavad Ehsani Ardakani

**Affiliations:** 1 *Faculty of Medicine, Ilam University of Medical Sciences, Ilam, Iran*; 2 *Faculty of Medicine, Shahid Beheshti University of Medical Sciences, Tehran, Iran*; 3 *Proteomics Research Center, Shahid Beheshti University of Medical Sciences, Tehran, Iran*; 4 *Basic and Molecular Epidemiology of Gastrointestinal Disorders Research Center, Research Institute for Gastroenterology and Liver Diseases, Shahid Beheshti University of Medical Sciences, Tehran, Iran.*; 5 *Gastroenterology and Liver Diseases Research Center, Research Institute for Gastroenterology and Liver Diseases, Shahid Beheshti University of Medical Sciences, Tehran, Iran.*

**Keywords:** Colon cancer, Interactome, Gene ontology, Hub-bottleneck nodes, Biomarker candidate

## Abstract

**Aim:**

Assessment of related genes to colon cancer to introduce crucial ones, was the aim of this research.

**Background:**

Colon cancer is one of the invasive colorectal diseases. This disease is preventable and manageable if it be diagnosed in early stage. The aggressive tools for its detection imply more investigation for new molecular diagnostic methods.

**Methods:**

Numbers of 300 genes from String database (SD) are analyzed via constructed Protein-protein interaction (PPI) network by Cytoscape software 3.4.0. Based on centrality parameters the main connected component of network was analyzed and the crucial genes were introduced. Cluster analysis of the network and gene ontology for the nodes of the main cluster revealed more details about the role of the key proteins related to colon cancer disease.

**Results:**

The constructed network was consisted of 300 genes which among them 68 genes were isolated and the 232 other genes formed the main connected component. Ten crucial genes related to colon adenocarcinoma were introduced that presented in cluster 1. Gene ontology analysis showed that cluster 1 is involved in 226 biological processes which are classified in 25 groups.

**Conclusion:**

In conclusion, results indicate that the identified key proteins play significant roles in colon adenocarcinoma. It may be possible to introduce a few diagnostic biomarker candidates for colon cancer disease.

## Introduction

 Colon cancer is one of the invasive colorectal cancers and second cause of death of patients with cancer (1). Many researchers are focused on molecular biology of colon cancer and provided valuable aspects of this cancer for better understanding of this disease than the other solid cancers (2). It is preventable and manageable in early stage. Colonoscopy is the common method for detection of colon cancer disease. However, this diagnostic tool is an aggressive method, there is no efficient and safe instrument for prognosis and diagnosis of colon cancer disease (3). Genetics plays significant role in incidence and advances of colon adenocarcinoma disease. Consequently, many genes are introduced that are involved in colon cancer disease. The studies indicate that gene expression changes for many of well-known genes are accompanied with onset of disease (4). Gene analysis and screening can provide useful prospective about molecular mechanism of diseases. Protein-protein interaction network recently is attracted attention of many scientists and researchers in medicine (5). The related genes of a certain disease are retrieved and analyzed under a precise and logical process in the interacted unit as a network. Each network contains many elements such as genes or proteins that call nodes and the links (edges) between them (6). Topological analysis of PPI network is a process that based on graph theory assesses network properties. Centrality parameters such as degree, betweenness centrality (BC), closeness centrality (CC) and stress are the valuable indices that discriminate the nodes in a network (7). Degree value refers to the numbers of edges that terminated to a node and high degree value for a node is corresponding to the hub node. BC is a function of the shortest paths that passes through a node and indicates to the control role of the node on the other nodes. The node with high value of BC is known as bottleneck node. Closeness the other function of shortest paths refers to speed of influence of information from the node to the other nodes. Stress of a node shows the numbers of the shortest paths that pass through that node (8-10). So these criteria are useful tools for ranking of the nodes of a network. There are many studies that analyzed molecular aspects of different diseases via the same methods (11-13). Gene ontology assesses biological processes, molecular functions and cellular components for a set of genes and can provide detail molecular information about them. The numerous diseases are analyzed via gene ontology (14, 15). Detection of the involved biochemical pathways in the diseases is a significant method for better understanding of molecular mechanism of incidence and advances in etiology of diseases (16, 17). Early detection and effective safe diagnosis of diseases require more investigation in the molecular aspects of diseases. The significant role of genetics in incidence and progress of diseases is an accepted rule in medicine. There are many evidences about the direct or indirect roles of a single or set of genes in a certain disease. Mutations and dysregulation of gene expression are accompanied with gross alterations in physiological and pathological conditions (18). Since the genetically findings are so dispersed and unorganized, suitable analytical methods are required for evaluation and validation of them. Protein-protein interaction analysis is used for interpretation of molecular aspects of the vast ranges of diseases. (19). Several gastrohepato diseases are evaluated via PPI network analysis and useful information are achieved (20, 21). The main aim of this paper is introducing a precise and restricted protein panel involved in the colon adenocarcinoma by analyzing the related genes via PPI network construction and gene ontology assessment. These proteins potentially can be considered as biomarker candidates for colon adenocarcinoma. 

## Methods

Cytoscape 3.4 is one of the free sources that can be used to provide related proteins to diseases. Cytoscape is compatible with different sources. This software and its applications are useful tools for data providing and analyzing via protein-protein interaction network. String Database (SD) (http://string-db.org/) is one of the efficient interaction sources that is available via Cytoscape (22, 23). Disease query is one of the three options of SD. In this research the related genes to colon adenocarcinoma that can construct a network were downloaded from disease query. When 100 or 200 genes were downloaded all of them involved in the network but among 300 genes only 232 ones constructed the network. So the 300 related genes to colon adenocarcinoma were analyzed via PPI network. The connected components of the constructed PPI network were identified. The centrality parameters of the main connected component were analyzed and the hub nodes were determined based on degree cut-off (Mean + 2 standard deviation) (24, 25). The top 5% of the nodes based on BC, CC and stress were chosen for more analysis (11). Distribution of degree, betweenness centrality and closeness centrality were considered for network analysis (26). Clustering has been used to provide more details of studied graph elements (27). Cluster analysis of the main connected component was done and the main cluster (cluster-1) was analyzed and its components were assessed. Gene ontology analysis (biological process) for the nodes of cluster-1 was done by the application of ClueGO. Based on attribution of at least three genes in a term and Term P-value, Term P-value corrected with Bonferroni step down, Group P-value and Group P-value corrected with Bonferroni step down≤0.001, the identified terms were grouped and analyzed for more resolution (28). 

## Results

**Figure 1:**
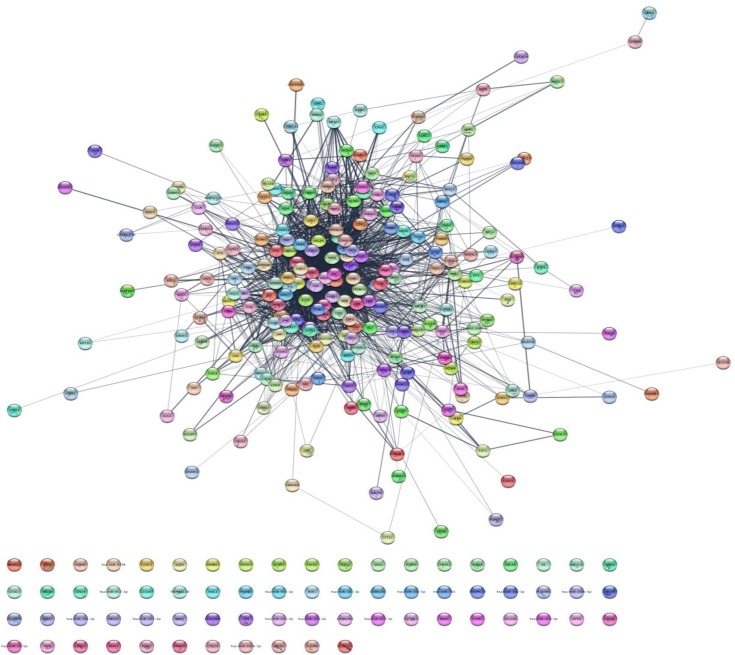
PPI network of colon adenocarcinoma. The network consists of 300 nodes, including 68 isolated nodes and 232 connected nodes. The main connected component includes 232 nodes and 2097 edges

**Figure 2:**
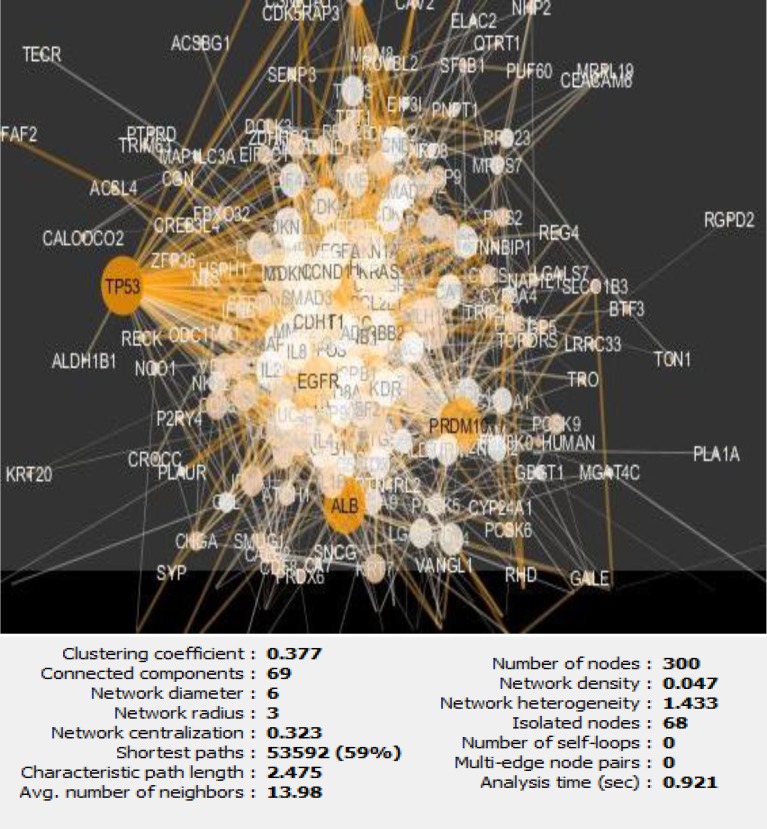
Main connected component of PPI network of colon adenocarcinoma. The 232 nodes are layout by degree value (The bigger and more dark circle correspond to the bigger value of degree

**Figure 3:**
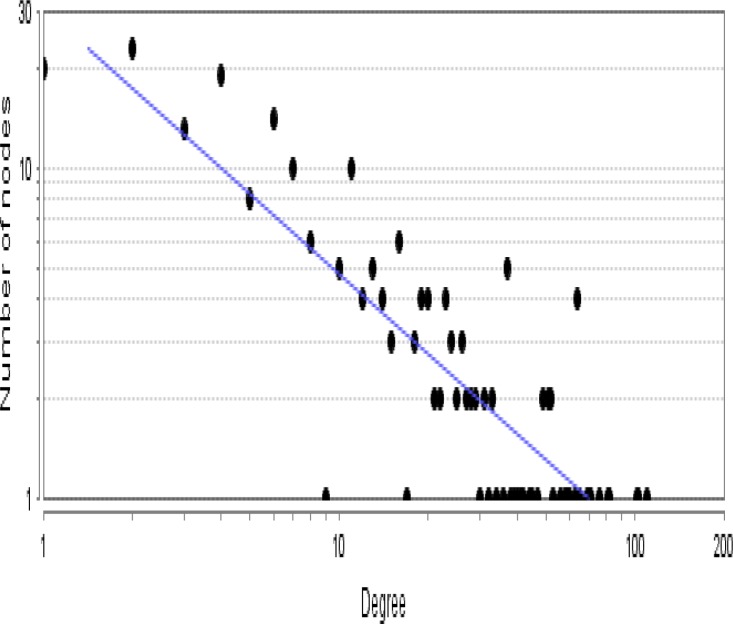
Degree distribution for the nodes of main connected component is presented. A power law y=30.370X^-0.866^ was fitted. Correlation: 0.866 and R-squared: 0.734 were obtained

**Figure 4.**
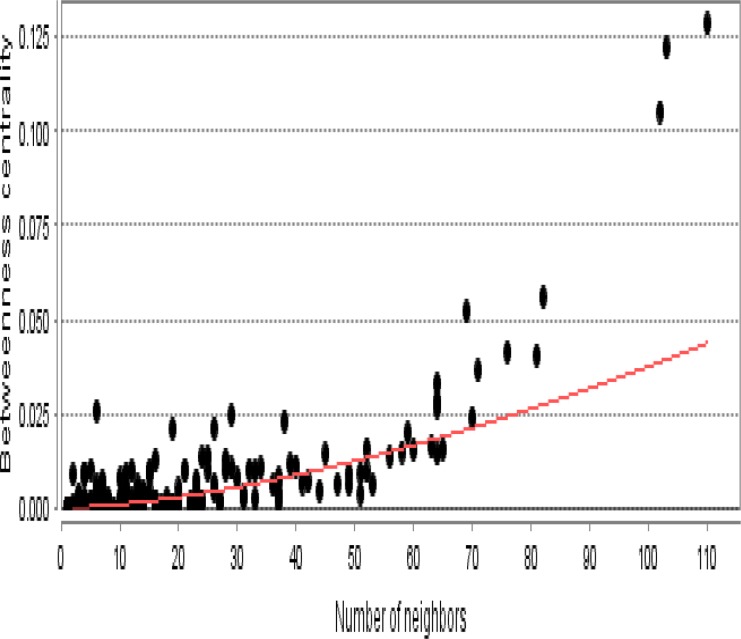
Betweenness centrality distribution for the main connected component is presented. A power law y=0.000X^1.587^ was fitted. Correlation: 0.858 and R-squared: 0.506 were obtained

**Figure 5.**
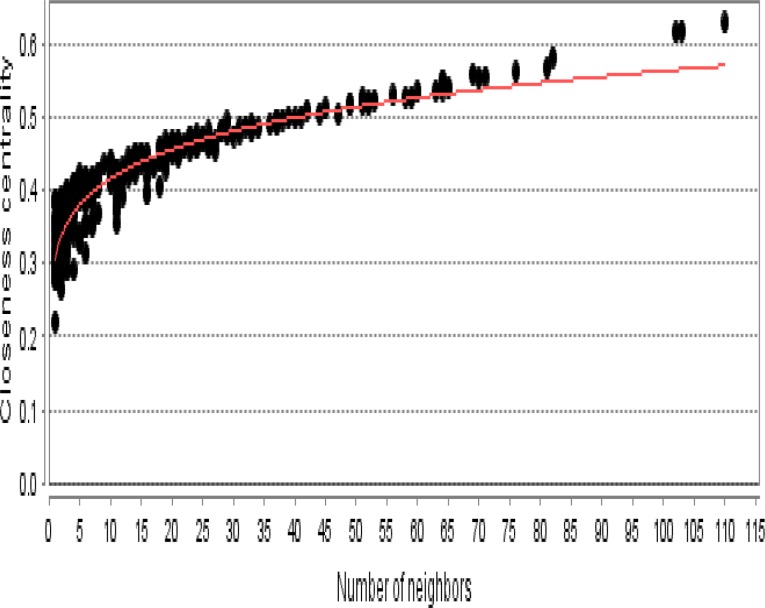
Closeness centrality distribution for the main connected component is presented. A power law y=0.308X^0.131^ was fitted. Correlation: 0.929 and R-squared: 0.821 were obtained

**Figure 6:**
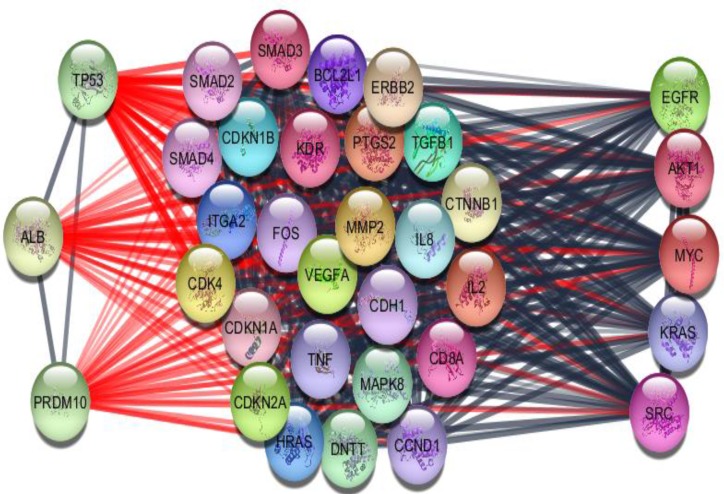
Cluster-1 includes 34 nodes and 512 edges. The left column (TP53, ALB and PRDM10) and the right column (EGFR, AKT1, MYC, KRAS and SRC) are the eight potent and moderate crucial genes respectively. All hub nodes and the weak crucial nodes (CDH1 and CTNNB1) are presented in this cluster

**Figure 7:**
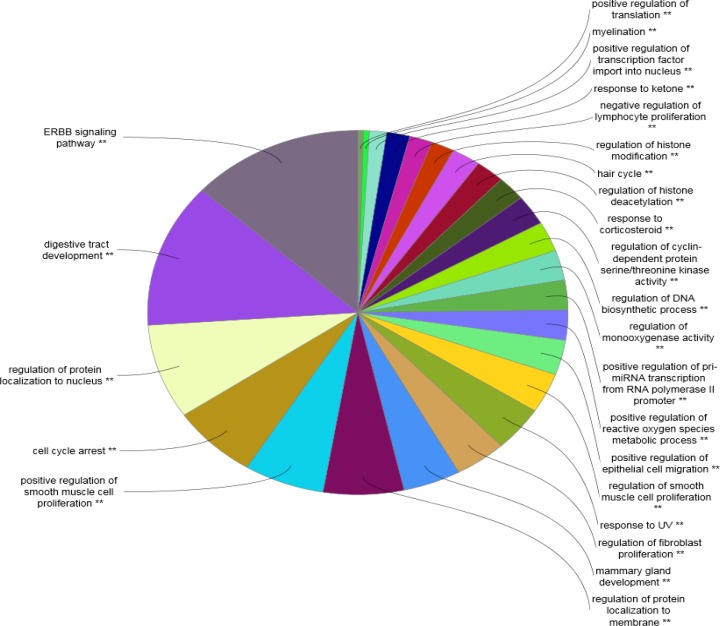
Biological processes relative to the nodes of cluster 1are grouped. Numbers of 226 terms are classified in 25 groups. Group P-value corrected with Bonferroni step down≤0.001

As it is shown in the figure 1 the constructed network includes 68 isolated nodes and a connected component of 232 nodes and 2097 edges. The nodes of main connected component of PPI network are layout by degree value (see figure 2). Distribution of edges between the nodes is not homogeny and the weight of the nodes (based on interaction with the other genes) is different. Since the nodes are layout by degree value more differentially details about the interacted nodes are appeared. Distribution of degree, betweenness centrality and closeness centrality (figures 3-5) are corresponded to the scale free network (29). Numbers of 16 top nodes based on degree value (the hub-nodes) and the top 5% of the nodes based on BC, CC and stress values are determined and tabulated in the table 1. As it is shown in the table 1, there are 11 hub-bottleneck nodes (The common nodes between the 16 hub nodes and the top 5% nodes based on betweenness value). As it is shown in table 1, all bottleneck genes except GUCY2C are hub nodes. The hub-bottleneck nodes that are presented in the both top 5% genes based on CC and stress (see table 1) are selected as crucial genes related to colon adenocarcinoma. These genes are tabulated in table 2. As it is depicted in the figures 3-5 and table 2, centrality parameters amounts for TP53, ALB and PRDM10 are extremely different from the other crucial nodes so can be considered as potent crucial genes. In the other hand CDH1 and CTNNB1 are the weak crucial nodes. The other five critical nodes are considered as moderate crucial genes. The finding indicates that the main connected component includes 11 clusters. Based on presence of the crucial nodes in the cluster, cluster 1 is the most important one. This cluster includes all crucial nodes and also 16 hub nodes (see figure 6). Since biological process (BP) is a useful tool to determine the role of an individual protein (30, 31), the BP analysis for the nodes of cluster-1 was done. The numbers of 226 terms were identified and categorized in 25 groups (see figure 7). 

## Discussion

Protein-protein interaction network analysis as a useful method is applied in the field of biomarker discovery of many diseases (32-35). In the present study 300 genes related to colon cancer are retrieved and assessed by PPI network. The numbers of 232 genes constructed an integrative network. 

Since these were extracted from databanks therefore their relationship with colon cancer is reported at least in one study. The heterogenic role of a network elements based on graph theory is discussed in several studies (5, 36). Here the genes are evaluated based on importance of their role in the network. Ten crucial genes which mostly interact with the other nodes of network and control them are introduced. This is a major advantage of network analysis that discriminates a few nodes among huge number of the nodes of a network (37). For better interpretation, the ten key genes are classified in three groups; the first group including TP53, ALB and PRDM10 as potent crucial genes, the second category (EGFR, AKT1, MYC, KRAS and SRC) as normal key genes and the last group (CDH1 and CTNNB1) as weak crucial nodes.

The role of TP53 and PRDM10 in colorectal cancers are discussed in details (38, 39). ALB expression change in colorectal cancers is reported in many documents (40). In addition expression change of these three genes in various cancers are confirmed and discussed in detail (41-43). Since sensitivity and specificity of a suggested biomarker are two important indices (44, 45), it seems that using each one as biomarker is not possible. 

Correlation between EGFR, AKT1, MYC, KRAS and SRC and, colorectal cancers separately or in combination with the other genes are studied and confirmed (46-49). As like the members of the first group, the role of these genes in development of the other cancers is reported. For example, the role of EGFR in non–small cell lung cancer and multiple cancer types are evaluated (50-53). Significant role of MYC in the various pathways of cancers is studied and confirmed (54, 55). Further analysis revealed that the ten highlighted genes and all hub genes (the listed genes in table 1) are presented completely in cluster 1 (see figure 6). Therefore, it seems that cluster 1 (including 34 nodes) is tightly related to colon adenocarcinoma. The finding indicates that this cluster is involved in 25 biological processes that mostly are related to cancer (see figure 7). ERBB (EGFR) signaling pathway is highlighted in figure 7. This pathway plays important roles in cell division control, cell motility and survival. ERBB activity changes are reported in a wide variety of human cancers (56). Significant relationship between this pathway and colorectal cancer is studied and discussed(57). As it is depicted in figure 7, digestive tract development is the second major biological process related to colon cancer. Occurrence of a wide verity changes in many biological process such as digestive tract development during colon cancer is accepted. The other major biological process is involved in proliferation, cell signaling and the other process related to cell cycle process. Indeed, the biological process emphases that the introduced cluster is a functional organization related to colon cancer.

The finding indicates that the introduced crucial genes are the affective and major elements in onset and progress human colon adenocarcinoma. As discussed expression change of these genes in various cancers is a big problem to use each of them as suitable biomarker related to colon cancer. Suggestion of several genes as biomarker panels in the case of certain dieses is a well-known established method (5, 58-61). So we suggest that expression change of these ten key genes in patients be evaluated for finding an affective biomarker panel of combination of few genes related to colon cancer. 

 PPI network analysis showed that there are ten crucial proteins including; TP53, ALB, PRDM10, EGFR, AKT1, MYC, KRAS, SRC CDH1 and CTNNB1 are related to colon adenocarcinoma disease. The role of the first three proteins is dominated relative to the last seven proteins. It can be concluded that this protein panel can be evaluated to achieve a useful tool in colon adenocarcinoma diagnosis. Screening of large numbers of genes to introduce few crucial related ones to colon adenocarcinoma is the main finding of this research.

**Table 1:** Numbers of 16 hub nodes and the top 5% nodes based on betweennness centrality (BC), closeness centrality (CC) and stress values are shown. The elements of the table are sorted by largest to smallest values of degree, BC, CC and stress

R	Hub genes	Bottleneck genes	Top 5% nodes based on CC	Top 5% nodes based on Stress
1	TP53	TP53	TP53	ALB
2	ALB	ALB	ALB	TP53
3	PRDM10	PRDM10	PRDM10	PRDM10
4	EGFR	EGFR	EGFR	EGFR
5	AKT1	SRC	AKT1	SRC
6	MYC	MYC	MYC	AKT1
7	KRAS	AKT1	SRC	KRAS
8	HRAS	KRAS	KRAS	MYC
9	SRC	CTNNB1	HRAS	HRAS
10	CCND1	CDH1	CDH1	CTNNB1
11	CDH1	ITGA2	CTNNB1	CDH1
12	ITGA2	GUCY2C	ERBB2	ITGA2
13	CTNNB1	-	-	-
14	ERBB2	-	-	-
15	FOS	-	-	-
16	TNF	-	-	-

**Table 2:** List of 10 crucial genes related to human colon adenocarcinoma PPI network. Betweennness centrality (BC), closeness centrality (CC), stress values and disease score are presented

**R**	**name**	**description**	**Degree**	**BC**	**CC**	**Stress**	**DS**
1	TP53	tumor protein p53	110	0.13	0.63	49586	1.9
2	ALB	albumin	103	0.12	0.62	50494	0.8
3	PRDM10	PR domain containing 10	102	0.11	0.61	47678	0.9
4	EGFR	epidermal growth factor receptor	82	0.06	0.58	27510	1.0
5	AKT1	v-akt murine thymoma viral oncogene homolog 1	81	0.04	0.57	22286	1.0
6	MYC	v-myc myelocytomatosis viral oncogene homolog (avian)	76	0.04	0.56	20724	1.4
7	KRAS	v-Ki-ras2 Kirsten rat sarcoma viral oncogene homolog	71	0.04	0.56	22076	1.7
8	SRC	v-src sarcoma (Schmidt-Ruppin A-2) viral oncogene homolog (avian)	69	0.05	0.56	23574	0.6
9	CDH1	cadherin 1, type 1, E-cadherin (epithelial)	64	0.03	0.55	15662	1.4
10	CTNNB1	catenin (cadherin-associated protein), beta 1, 88kDa	64	0.03	0.55	16936	1.7

## Conflict of interests

The authors declare that they have no conflict of interest.
